# Nanoscale Peptide Self-assemblies Boost BCG-primed Cellular Immunity Against *Mycobacterium tuberculosis*

**DOI:** 10.1038/s41598-018-31089-y

**Published:** 2018-08-21

**Authors:** Charles B. Chesson, Matthew Huante, Rebecca J. Nusbaum, Aida G. Walker, Tara M. Clover, Jagannath Chinnaswamy, Janice J. Endsley, Jai S. Rudra

**Affiliations:** 1 Department of Surgical Oncology, Rutgers Cancer Institute of New Jersey, New Brunswick, NJ 08823 USA; 2 Department of Microbiology and Immunology, University of Texas Medical Branch, Galveston, TX 77555 USA; 3 Department of Pathology, School of Dental Medicine, University of Pennsylvania, Philadelphia, PA 19104 USA; 4 Department of Pharmacology and Toxicology, University of Texas Medical Branch, Galveston, 77555 Texas USA; 5 Department of Pathology and Laboratory Medicine, McGovern Medical School, University of Texas Health Science Center at Houston, Houston, TX 77030 USA; 6 Sealy Institute for Vaccine Sciences, University of Texas Medical Branch, Galveston, TX 77555 USA

## Abstract

Bacillus Calmette-Guerin (BCG) is the only vaccine against TB and has limited protection efficacy, which wanes past adolescence. Multifunctional CD8+ T cells (IFN-γ+/TNF-α+/IL-2+) are associated with lower reactivation risk and enhanced control of active *Mtb* infection. Since boosting with BCG is contraindicated, booster vaccines that augment T cell immunity in the lungs of BCG-vaccinated individuals are urgently needed. We developed a vaccination strategy based on self-assembling peptide nanofibers presenting *Mtb*-specific CD8+ or CD4+ T cell epitopes that induce high frequency and antigen-specific effector memory T cells producing IFN-γ and IL-2. Intranasal immunization with peptide nanofibers was well tolerated in mice leading to increased antigen-specific CD8+ T cell population in the lungs. Co-assembled nanofibers of CD8+ T cell epitopes and toll-like receptor 2 (TLR2) agonists induced a 8-fold expansion in multifunctional CD8+ T cell populations in the lungs of vaccinated mice. Aerosol challenge with *Mtb* in BCG-primed and nanofiber-boosted mice provided an additional 0.5-log CFU reduction in lung bacterial load and indicating enhanced protection compared to BCG alone. Together, these data suggest that heterologous prime-boost with BCG and peptide nanofiber vaccines induces cell mediated immunity in the lung, reduces bacterial burden, and is a potentially safer alternative for boosting BCG-primed immunity.

## Introduction

Lung infection with *Mycobacterium tuberculosis* (*Mtb*), the causative agent of pulmonary tuberculosis (TB), is a major global health concern with nearly 10.4 million infections and 1.67 million deaths reported in 2016 and has surpassed HIV/AIDS as the leading cause of mortality worldwide^1^. *Mtb* is transmitted via aerosol droplets that facilitate access to the lung and infection of alveolar macrophages. Lung-specific immunity mediated by CD8^+^ and CD4^+^ T cells, is necessary for host defense against TB. Multifunctional CD8^+^ T cells that produce IFN-γ/TNF-α/IL-2 have been associated with lower risk of reactivation and enhanced control of active *Mtb* infection^2^. Since 1921, Bacillus Calmette-Guérin (BCG) is the only approved vaccine against *Mtb* and an estimated 1 billion people have received it worldwide. However, BCG has important limitations including the waning of protection beyond adolescence, safety concerns for use in immunocompromised individuals, and lack of efficacy to boost existing immunity^3^. Since boosting with BCG is poorly efficacious, subunit vaccines that incorporate protective CD8^+^ and CD4^+^ T helper epitopes, which augment cellular immunity in the lung of BCG-vaccinated individuals, are urgently required.

In recent years, applications of nanotechnology in the field of immunology and vaccine development has led to the development of nanomaterials-based strategies for targeted or sustained delivery of antigens and immunomodulators for improving CD8^+^ T cell immunity against infectious and non-infectious diseases^4^. Notably, platforms based on polymeric micro and nanoparticles^5^, cross-linked multi-lamellar vesicles^6^, self-assembling peptides^7,8^, peptide amphiphiles^9^, and multilayer thin films^10,11^ have shown considerable promise in preclinical studies. We have previously reported that *de novo* designed short peptides that assemble into β-sheet rich nanofibers in physiological buffers are potent immunostimulants and induce robust antibody and CD4^+^ T helper cell responses against conjugated subunit antigens^12–16^. The ability of peptide nanofiber vaccines to elicit protective antibody responses has been demonstrated in mouse models of Herpes Simplex Virus^17^, West Nile^18^, cancer^19^, and cocaine addiction^20^. Our previous investigations using model MHC class I peptide antigen, OVA (chicken egg ovalbumin, aa 257–2644), have demonstrated that parenteral vaccination with nanofiber vaccines elicits robust effector and memory CD8^+^ T cell responses and protects mice from infection with transgenic influenza virus expressing OVA^8^. However, the strong CD8^+^ T cell responses elicited against highly immunogenic OVA peptide may not be indicative of responses to more native epitopes from pathogens such as *Mtb*. Furthermore, the microenvironment and cellular populations of mucosal lymphoid tissues differ from peripheral lymph nodes and are therefore distinct as an inductive site for priming of acquired immune responses^21^. Thus, the function of peptide nanofiber vaccines to elicit mucosal CD8^+^ Tcell immunity and protect against *Mtb* infections needs to be defined.

In this work we investigated the ability of peptide nanofiber vaccines bearing *Mtb*-specific epitopes to induce systemic or lung-resident cellular immune cell responses following parenteral or intranasal vaccination. Exploiting the modular nature of self-assembling peptides, we further developed nanofiber formulations combining *Mtb*-specific CD8^+^ T cell epitopes in combination with CD4^+^ T helper epitopes or toll-like receptor (TLR) agonists and investigated the ability of co-assembled formulations to enhance cellular immunity via the expansion of multifunctional CD8^+^ T cells (CD8^+^ T cells that produce IFN-γ/TNF-α/IL-2) in the lung. We also investigated vaccination efficacy in BCG-primed mice following an aerosol challenge with *Mtb*. Our data indicate that self-assembling peptides bearing *Mtb*-specific antigens can elicit lung-specific mucosal immune responses and co-assembled formulations of *Mtb* antigens and TLR2 agonists can boost BCG-primed immunity and protect against *Mtb* infection.

## Results

### Identification of Mtb epitopes and synthesis of self-assembling peptide nanofiber vaccines

**Figure 1:**
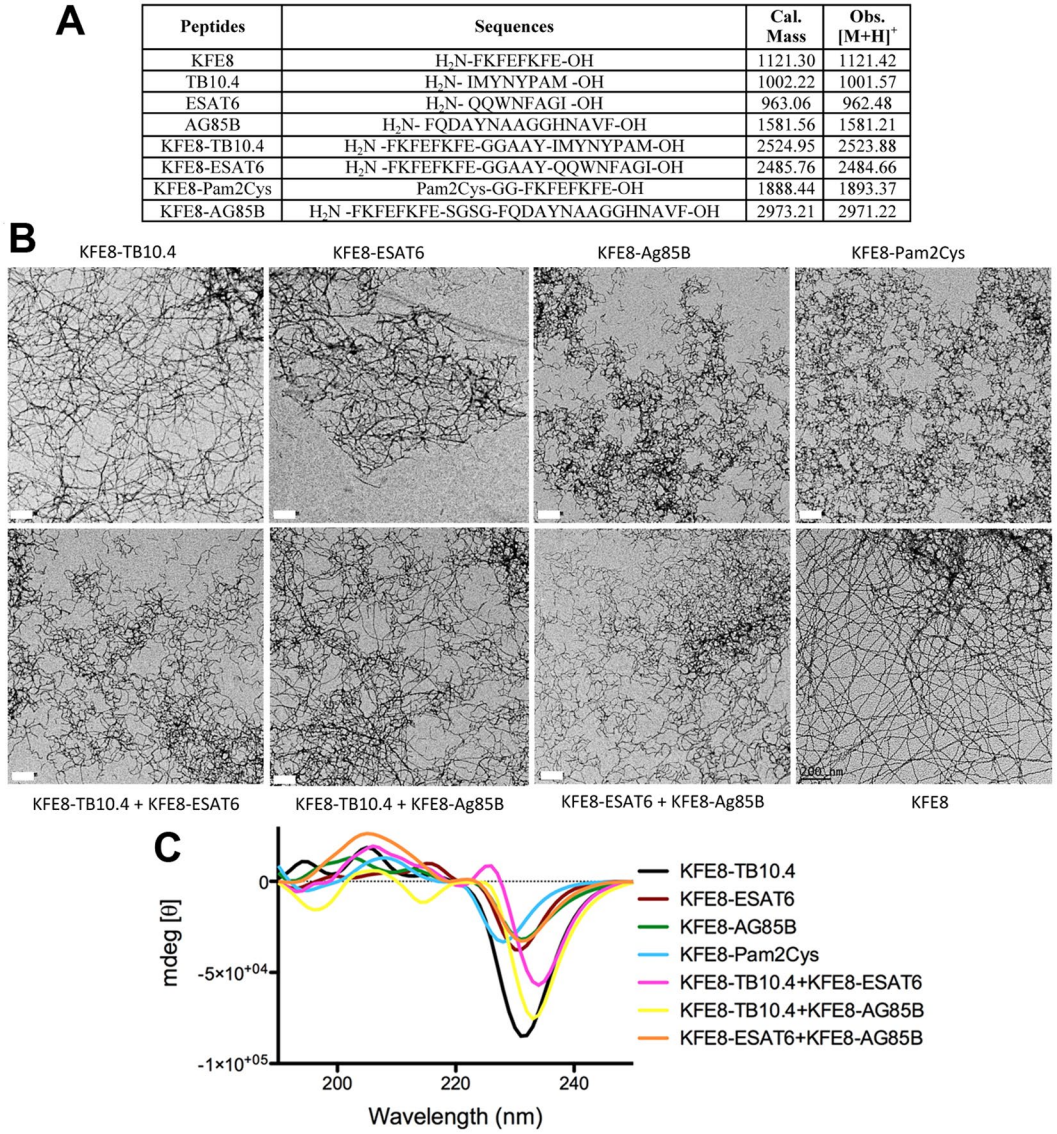
Peptide sequences synthesized for use in this study including the corresponding observed MALDI-MS [M + H] compared to the expected mass (**A**). Transmission electron microscopy (TEM) images of single and co-assembled epitope formulations (**B**). Scale bar is 200 nm. Circular dichrosim spectra for single and co-assembled epitopes displaying a peak minima at 200–230 nm indicative of β-sheet structure of the nanofiber formulations (**C**).

Our goal was to identify strong *Mtb*-specific antigen targets that could be conjugated to self-assembling peptides and tested in a heterologous prime-boost regimen with BCG. Our search of the literature yielded *Mtb* protein antigens TB10.4 (Rv0288), ESAT6 (early secretory antigenic target gene 6), and antigen 85 Complex B (Ag85B) as the most studied and strongly immunogenic *Mtb* proteins in animal models^22–24^. We further selected several immunodominant epitopes from Ag85B, TB10.4, and ESAT6 that have been shown to have strong binding to H-2k^b^ (C57BL6) or H-2k^d^ (BALB/c) MHC class I or class II and also reactivity with leukocytes from human donors with immune memory to mycobacterial proteins for improved translational potential. The best epitopes from literature were identified as CD8^+^ T cell epitopes IMYNYPAM (from TB10.4) and QQWNFAGI (from ESAT6) and CD4^+^ T cell epitope FQDAYNAAGGHNAVF (from Ag85B). These peptides were synthesized in tandem with the self-assembling peptide domain KFE8 (FKFEFKFE) using the spacer sequence GGAAY that facilitates cleavage by endosomal proteases in the antigen-presenting cell^25^. Throughout the manuscript we will refer to the epitopes by their proteins of origin for simplicity (Fig. 1). Secondary structure and self-assembly of *Mtb* epitope-KFE8 conjugates were evaluated using circular dichroism spectroscopy and transmission electron microscopy (TEM). Microscopy data indicated that antigen-KFE8 conjugates assembled into nanofibers and CD spectra indicated a β-sheet secondary structure for epitope-bearing self-assembling peptides as observed by us previously^26^.

### Nanofibers bearing Mtb CD8+ T cell epitopes from TB10.4 or ESAT6 are immunogenic as single formulations or when co-assembled

**Figure 2:**
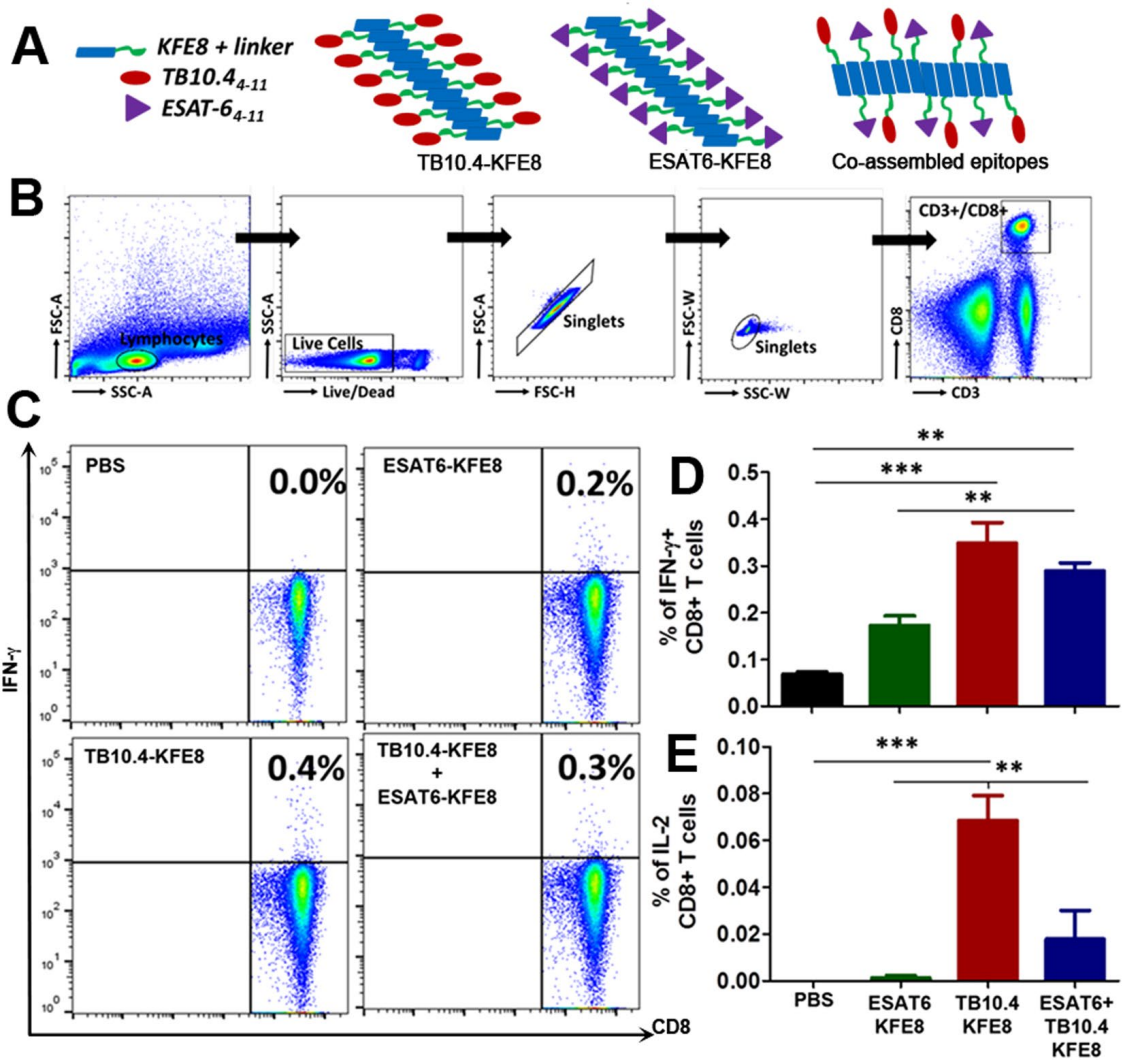
Co-assembled nanofibers bearing *Mtb*-specific CD8+ T cell epitopes elicit antigen specific immune responses. Schematic shows self-assembly of single or dual epitope nanofiber formulations (**A**). Gating strategy used for identification of activated CD8+ T cells isolated from draining lymph nodes 30 days post-immunization (**B**). Representative flow plots for IFN-γ+ CD8+ T cells in mice vaccinated with single epitope or dual epitope formulations (**C**). Mean percentages ± SEM of IFN-γ+ CD8+ T cells (**D**), and IL-2+ CD8+ T cells (**E**) measured *ex vivo* in lymph nodes of mice vaccinated with TB10.4 or ESAT6 nanofibers or co-assembled nanofibers. Data is representative of two independent experiments with similar results and ***p < 0.001, **p < 0.01 by one-way ANOVA with multiple comparisons of means.

The modular nature of self-assembly allows for simple mixing of peptides with different functional moieties linked to the self-assembling domain to generate multifunctional biomaterials (Fig. 2A)^27^. This property has been exploited for generating self-assembling peptide scaffolds that display multiple ECM signals, antigens, and small chemical moieties for enhancing cell attachment and proliferation, controlling matrix stiffness, and fine-tuning the ratio of humoral and cellular immune responses^28,29^. Co-assembled nanofiber vaccines bearing two different antigens have been shown to result in dual antibody production in mice without loss of immunogenicity^14^. However, whether co-assembled formulations of CD8+ T cell epitopes can lead to antigen-specific CD8+ T cell activation and cytokine production is not known. To address this, mice were primed and boosted 30 days apart with KFE8 nanofibers bearing either TB10.4 or ESAT6 or co-assembled nanofibers (1:1) and antigenicity was measured using flow cytometric detection of intracellular cytokine production following *ex vivo* stimulation of lymphocytes with soluble peptide antigens (gating strategy shown in Fig. 2B). Data indicated that vaccination with TB10.4-KFE8 nanofibers led to significantly increased numbers of IFN-γ+ and IL-2+ CD8+ T cells in vaccinated mice. Immunization with ESAT6-KFE8 nanofibers only induced moderate levels of IFN-γ+, and poor IL-2+ CD8+ T cells compared to TB10.4-KFE8 (Fig. 2C–E). The ESAT6 epitope identified by predictive binding strength to H-2k^b,d^ MHC class I is nested within a well-characterized CD4^+^ T cell epitope of ESAT6 (MTE*QQWNFAGI*EAAASAIQG). Other studies have demonstrated that while the peptide binds to MHC class I with nanomolar affinity (IC_50_ ~180 nM), it is poorly immunogenic as measured by antigen-specific IFN-γ production following vaccination^30^. Lymphocytes from mice receiving co-assembled nanofibers of ESAT-6 and TB10.4 were stimulated by both peptides and data show that cytokine responses were less robust than those induced by TB10.4 epitope alone. This suggests that TB10.4 is probably a more potent antigen compared to ESAT6 and that antigenic competition may subdue cytokine activation following delivery of co-assembled formulations. Nonetheless, the data do suggest that co-assembled peptide nanofibers bearing multiple CD8+ T cell epitopes can be fabricated and activate antigen-specific cytokine activation. Further optimization of co-assembly formulations may identify strategies to present diverse antigens that enhance the breadth of protection.

### CD4+ T helper epitopes stimulate robust recall responses when co-assembled with CD8+ T cell epitopes

CD4^+^ T helper cells are critical for controlling *Mtb* infection through Th1 cytokine expression that activates macrophage bactericidal function and regulates the actions of other lymphocytes including CD8+ T cells^31^. Clinical data show that in patients with controlled *Mtb* infection, a high proportion of CD4^+^ T cells are responsive to the secretory protein Ag85B^32–34^. To assess potentiation of CD8+ T cell responses when co-assembled with CD4+ T helper epitopes, we synthesized Ag85B-KFE8 to target CD4+ T cells and co-assembled it with TB10.4-KFE8 or ESAT6-KFE8 (1:1) formulations that target CD8+ T cells. Mice were primed with co-assembled peptide nanofibers bearing TB10.4 and ESAT6 (1:1) or TB10.4 or ESAT6 in combination with Ag85B (1:1) and boosted 90 days later. Intracellular cytokine production by CD8+ T and CD4+ T cells was assessed following *ex vivo* stimulation of lymphocytes with the cognate antigens. To ensure that cytokine production was antigen specific, lymphocytes were also stimulated with irrelevant peptides (*e*.*g*. lymphocytes from mice vaccinated with nanofibers bearing ESAT6 and Ag85B were stimulated with TB10.4).

**Figure 3:**
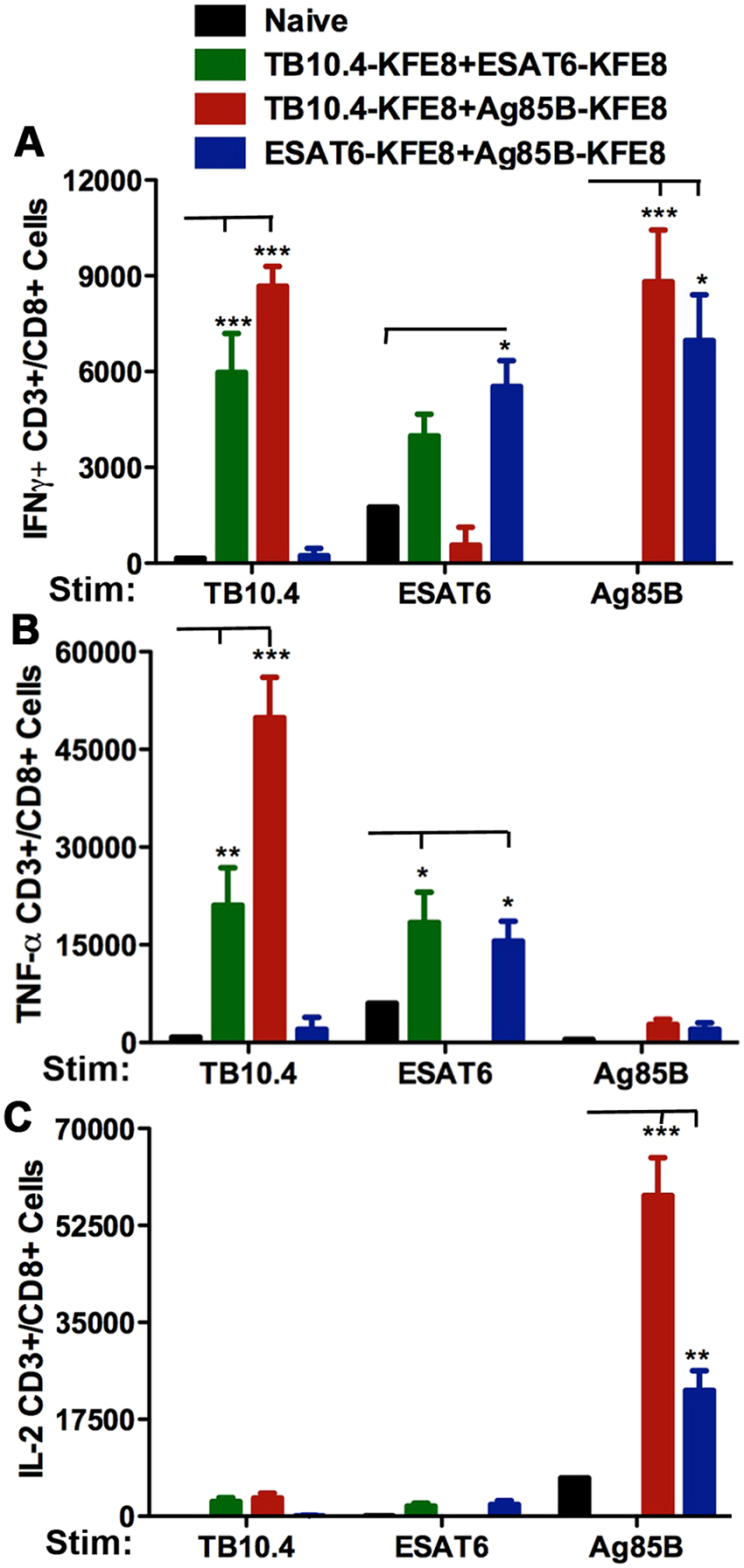
Co-assembled nanofiber vaccines bearing *Mtb*-specific CD8+ and CD4+ T cell epitopes stimulate robust recall responses. In mice vaccinated with co-assembled nanofibers of TB10.4-Ag85B (CD8-CD4) or ESAT6-Ag85B (CD8-CD4), stimulation with TB10.4, ESAT6, or Ag85B (lymphocytes isolated from popliteal lymph node 90 days post immunization) leads to increased numbers of CD8+ T cells producing IFN-γ, compared to mice vaccinated with TB10.4-ESAT6 (CD8-CD8) nanofibers (**A**). No differences in numbers of IL-2 producing CD8+ T cells were observed in mice vaccinated with TB10.4-Ag85B or ESAT6-Ag85B nanofibers when stimulated with TB10.4 or ESAT6. A significant increase in numbers of IL-2+ and IFN-γ+ CD8+ T cells was observed following stimulation with Ag85B, suggesting activation of antigen-specific helper function by Ag85B-specific CD4+ T cells. The increase in IL-2+ CD8+ T cells was also significantly higher when TB10.4 was co-delivered with Ag85B compared to ESAT6 (**C**). N = 5 mice per group and ***p < 0.001, **p < 0.01 and *p < 0.05 by two way ANOVA using Bonferroni post hoc test. Calculated p-values are compared to naïve control for each stimulation.

Results demonstrated the development of antigen-specific IFN-γ, TNF-α, and IL-2 recall responses by CD8^+^ T cells from lymph nodes of mice 90 d post-immunization (Fig. 3). Of note, data indicate even higher levels of IFN-γ+ and TNF-α+ CD8+ T cells in mice receiving TB10.4 or ESAT6 co-assembled with Ag85B as compared to the CD8+ T cell epitopes only (Fig. 3A,B). IFN-γ+ producing CD8+ T cells were lower in mice immunized with ESAT6 even in the presence of Ag85B compared to TB10.4 (Fig. 3A,B). The majority of cytokine positive cells within the TB10.4 co-assembled groups explicitly expressed IFN-γ, yet there was a trend towards co-expression of IL-2 within these groups (Figure S5). Interestingly, IFN-γ+ and IL-2+, but not TNF-α+ producing, CD8+ T cells were also observed when lymphocytes from mice vaccinated with TB10.4-Ag85B nanofibers or ESAT6-Ag85B nanofibers where stimulated *ex vivo* with the Ag85B CD4 epitope (Fig. 3A,C). These observations are consistent with the known helper function of CD4+ T cell produced cytokines such as IL-2 to activate bystander cells and amplify the antigen-specific immune response. IL-2 is a strong inducer of IFN-γ, and provides positive feedback to further induce IL-2, by both CD4+ and CD8+ T cells.

**Figure 4:**
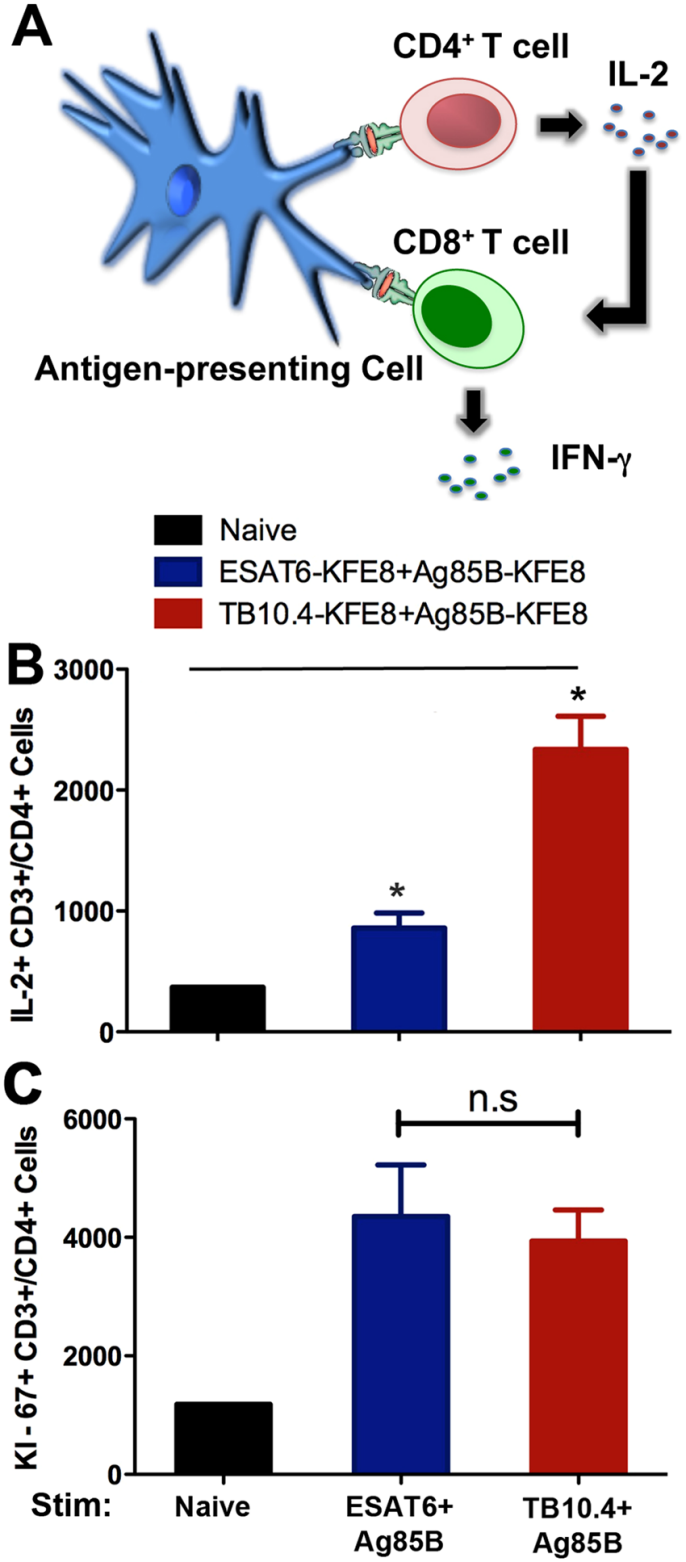
Co-assembly of CD4+ T cell epitopes (Ag85B) with CD8+ T cell epitopes (TB10.4 or ESAT6) induces robust CD4+ T cell recall responses. Schematic depicts helper function of cytokines produced by CD4+ T cells (**A**). Total numbers of single positive cells for IL-2 (**B**) and the proliferation marker Ki-67 (**C**) following *ex vivo* stimulation of lymphocytes isolated from popliteal lymph node 90 days post immunization with Ag85B in mice vaccinated with co-assembled nanofibers of ESAT6-Ag85B or TB10.4-Ag85B. Higher numbers of IL-2-producing CD4+ T cells were detected in co-assembled formulation of TB10.4-Ag85B compared to ESAT6-Ag85B, while proliferative responses were similar in both vaccine groups. *p < 0.05, by ANOVA by Tukey**’**s post-hoc test.

Data in Fig. 4 support this postulate and demonstrate antigen-specific activation of IL-2 and lymphoproliferation of CD4+ T cells from immunized mice after exposure to specific antigen. Intracellular cytokine staining of CD4+ T cells in mice indicated significantly less production of IL-2 by CD4+ T cells from mice receiving ESAT6-Ag85B nanofibers compared to mice vaccinated with TB10.4-Ag85B nanofibers (Fig. 4B). However, despite lower numbers of cytokine-producing CD4+ T cells, the proliferative response of CD4+ T cells was robust and similar in both ESAT-6 and TB10.4 vaccinated groups (Fig. 4C). Thus, the increased numbers of IFN-γ and IL-2 producing CD8+ T cells observed following *in vitro* exposure of splenic preparations to Ag85B (Fig. 3A,C) is likely due to helper function (e.g. IL-2) of *Mtb*-specific CD4+ T cell memory pools (Fig. 4A–C). Taken together the data indicate that the ESAT6 epitope has minimal immunogenicity as a single formulation, but diminishes multi-functional cytokine activation when co-delivered with other CD8+ or CD4+ T cell antigens. Stimulation with irrelevant peptides did not lead to any cytokine production across the groups tested confirming minimal bystander activation and antigen-specific recall responses (Fig. 3). However, it is not known whether co-assembly of CD4+ T cell epitopes with CD8+ T cell epitopes is required for robust CD4+ T cell responses observed here. Control studies investigating CD4+ T cell responses following vaccination with TB10.4 and ESAT6 nanofibers without Ag85B will be useful to address such claims. Overall, peptide nanofiber-based vaccines bearing CD8+ T cell and CD4+ T cell epitopes are immunogenic and elicit robust cellular immune responses following vaccination. Interestingly a strong antibody response directed towards the Ag85B peptide epitope was also observed in mice vaccinated with co-assembled nanofibers of TB10.4-Ag85B or ESAT6-Ag85B (Fig S1). It is not known whether Ag85B-specific antibodies play a role in the attenuation of the infection severity or promotion of latent infection. Our subsequent studies reported here will utilize TB10.4 as the CD8+ T cell and Ag85B as the CD4+ T cell epitope.

### Co-assembled nanofibers of toll-like receptor 2 (TLR2) agonist and TB10.4 enhance APC function of DC and increase multifunctional CD8+ T cell populations in the lung

**Figure 5:**
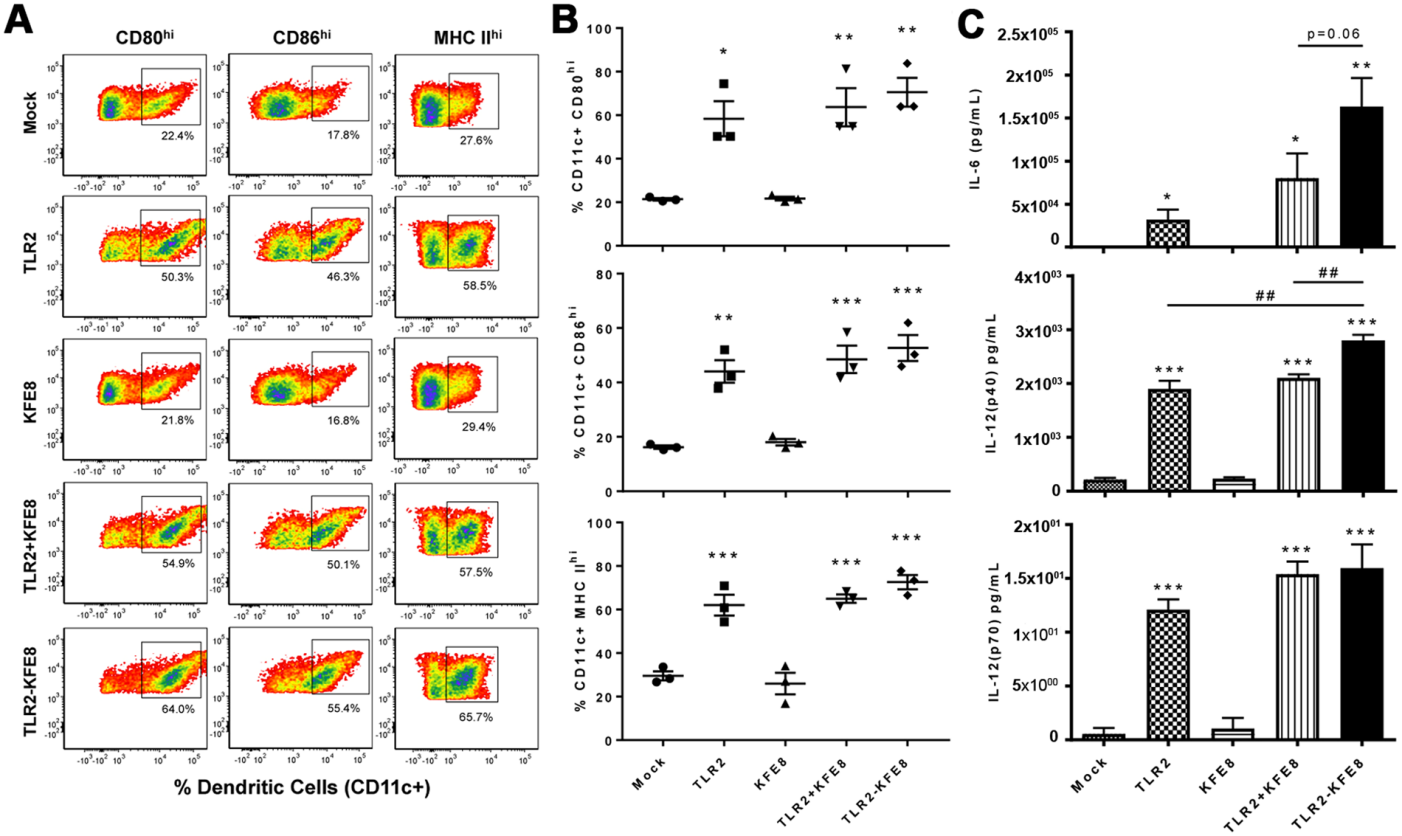
Pam2Cys-KFE8 nanofibers augment the antigen presenting cell function of dendritic cells. DCs were derived from bone marrow and treated for 24 h with BSA (mock), Pam2Cys (TLR2), NF (KFE8), NF and Pam2Cys (KFE8 + TLR2) or NF conjugated to Pam2Cys (TLR2-KFE8). (**A**) Flow cytometric analysis of a representative DC culture, and (**B**) summarized flow cytometry data from 3 animals, demonstrates that NF alone do not activate DCs while inclusion of a TLR2 agonist markedly increases expression of markers (CD80, CD86, and MHC II) associated with enhanced APC function. (**C**) Production of soluble cytokines, measured by ELISA, in DC culture supernatants demonstrate a TLR-2 dependent increase in IL-6 and IL-12 and suggest that direct conjugation of NF to Pam2Cys enhance these effects. Statistically significant differences compared to mock are indicated by, *p < 0.05, **p < 0.01, and ***p < 0.001 by ANOVA with Tukey’s post-hoc test. Similarly, # indicates differences between individual treatment groups.

In studies to date, peptide nanofiber vaccines have been used without added adjuvants and we recently reported that the immunogenicity of peptide nanofiber vaccines is due to enhanced macroautophagy-mediated antigen presentation^35^. However, the nanofibers themselves do not activate APCs similar to adjuvants that activate pathogen pattern recognition receptors such as TLR agonists that provide additional signals that direct T cell memory development^36,37^. Mice deficient in TLR2 have increased susceptibility to *Mtb* infection and TLR2 agonists have been shown to boost systemic and pulmonary immunity induced by *Mtb* subunit vaccines^38,39^. To improve immunogenicity of peptide nanofiber vaccines, we synthesized a self-assembling analog of TLR2 by conjugating the lipopeptide Cys((RS)−2,3-di(palmitoyloxy)-propyl)-OH (Pam2Cys) to the N-terminus of KFE8 and co-assembled it with TB10.4-KFE8. First, to assess whether Pam2Cys retains its TLR2 activation potential after conjugation to KFE8, bone marrow derived DCs were stimulated *in vitro* with Pam2Cys or Pam2Cys-KFE8 nanofibers for 48 h and cytokine production was evaluated. Untreated, or cells treated with bare KFE8 nanofibers were used as controls. Results indicated that similar to Pam2Cys alone, Pam2Cys-KFE8 nanofibers were able to activate CD11c + DCs leading to up-regulation of co-stimulatory molecules and cytokines (Fig. 5) characteristic of augmented APC function. CD80, CD86 and MHC class II were all strongly up-regulated after exposure to Pam2Cys nanofibers compared to controls (Fig. 5A). Production of pro-inflammatory cytokines IL-6 and IL-12 was detected in cells treated with Pam2Cys-KFE8 nanofibers similar to Pam2Cys (Fig. 5B), consistent with the effects of TLR2 activation. As expected, bare KFE8 nanofibers had no effect on expression of co-stimulatory molecules or cytokines. Further, no significant difference in activation was observed when Pam2Cys was simply admixed with bare KFE8 nanofibers or physically conjugated to KFE8 (Fig. 5A). Prior to vaccinating with co-assembled TB10.4-Pam2Cys nanofibers, the effect of Pam2Cys dosing on the number of antigen-specific cells was assessed by mixing the TB10.4-KFE8 and Pam2Cys-KFE8 in stoichiometric proportions. No significant differences between the numbers of TB10.4-specific CD8+ T cells were observed at Pam2Cys:TB10.4 ratio of 0.05:1, 0.1:1, and 0.25:1 (Fig. S2). In this study, we used a ratio of 0.2:1 (Pam2Cys:TB10.4).

**Figure 6:**
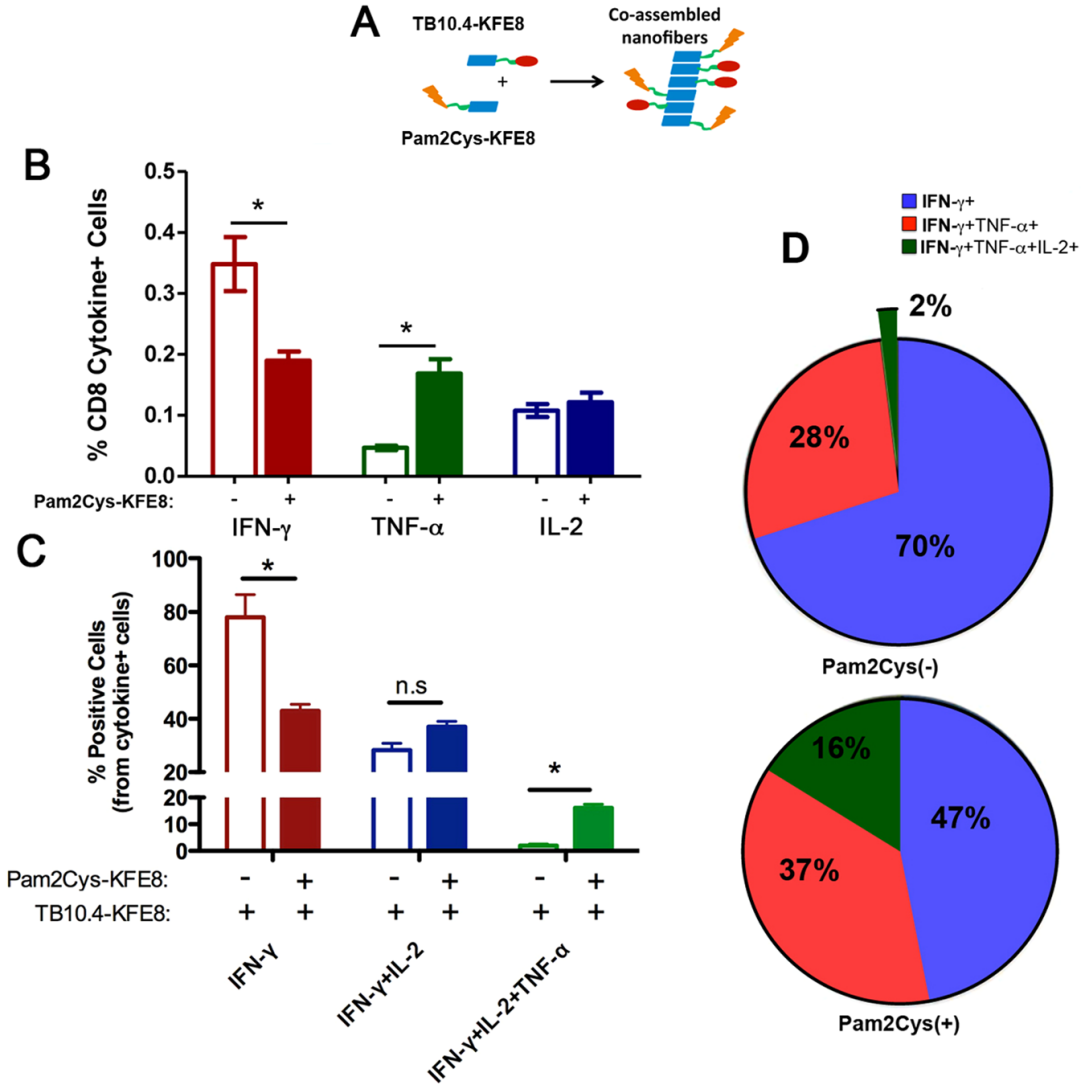
Co-assembled nanofibers of TB10.4 and TLR2 agonist increase the frequency of polyfunctional CD8+/CD3+ T cells. Schematic shows co-assembly of of TLR-KFE8 and TB10.4-KFE8 into single formulation with a representative electron micrograph (**A**). Lymphocytes were isolated from mediastinal lymph nodes lungs post immunization (foot pad prime and intranasal boost) and stimulated with TB10.4 peptide for 6 hours to induce cytokine expression. Total percentage for each cytokine IFN-γ, TNF-α, and IL-2 from the total population of CD8+/CD3+ T cells is shown. (**B**) Total percentage of single, double, or triple cytokine positive cells from all cytokine positive cells in the presence or absence of Pam2Cys-KFE8 is shown in panel (**C**). Multifunctional cell populations are significantly higher when Pam2Cys-KFE8 is co-assembled with TB10.4-KFE8. Pie charts are indicate the mean percentages of IFN-γ+ cells that also expressed IL-2, and TNF-α (**D**). N = 4 per group (**B**–**D**), *p < 0.05 by ANOVA using Tukey post hoc comparison.

Since *Mtb* enters the respiratory mucosa via inhalation of infectious droplets, lung-specific cell-mediated immunity has been shown to be necessary for host defense. BCG is administered only via the intradermal route in the clinic, therefore mucosal administration of subunit booster vaccines may confer a protective advantage due to the compartmentalized nature of the mucosal immune system. To test this, we primed mice in the footpad and boosted intranasally with TB10.4 nanofibers alone or co-assembled with Pam2Cys-KFE8 and assessed cytokine production by CD8+ T cell populations in the lung draining lymph nodes (mediastinal). Our data indicated that incorporation of Pam2Cys with TB10.4 leads to an 8-fold increase in the percentage of multifunctional CD8+ T cells (CD8^+^ T cells that produce IFN-γ/TNF-α/IL-2) compared to TB10.4 alone (Fig. 6). Interestingly, the production of TNF-α was significantly enhanced when Pam2Cys was added, though this observation was accompanied by a corresponding decrease in IFN-γ (Fig. 6A). The % of IL-2 producing CD8+ T cells, however, remained constant in the presence of Pam2Cys. Overall, the result of Pam2Cys incorporation with TB10.4 was augmented APC function of DC and generation of antigen-specific CD8^+^ T cells with broader poly-functional cytokine repertoires in the lung.

### Intranasal boost with co-assembled nanofibers of TB10.4 and Pam2Cys following BCG-prime enhances protection against Mtb challenge

**Figure 7:**
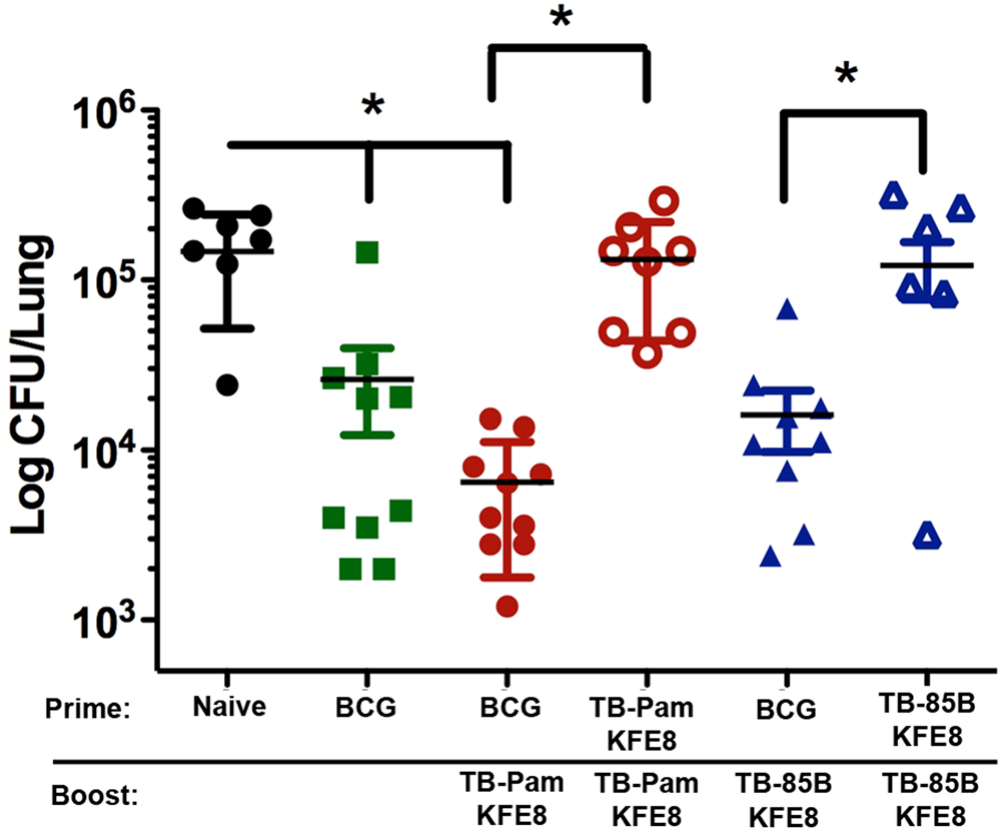
Boosting BCG-primed mice with peptide nanofiber vaccines leads to reduction of lung bacterial load. CFU enumeration data indicated significantly reduced lung bacterial loads and reduced liver dissemination (data not shown) in mice treated with BCG or primed with BCG and boosted with co-assembled nanofibers of Pam2Cys-KFE8 and TB10.4-KFE8 compared to untreated mice. Boosting with co-assembled nanofibers of Pam2Cys-KFE8 and TB10.4-KFE8 led to an additional 0.5 log_10_ CFU reduction in lung bacterial load compared to BCG alone. Prime-boost with peptide nanofiber vaccines in the absence of BCG priming did not lead to any reduction in lung bacterial load, as expected. *p < 0.05, by ANOVA with Tukey**’**s post-hoc test.

To assess whether intranasal administration of peptide nanofiber vaccines can boost BCG-primed immunity, mice were boosted intranasally with TB10.4 nanofibers alone or co-assembled with Ag85B or Pam2Cys 30 days after a BCG prime. Vaccinated and control animals were challenged with *Mtb* (H37Rv strain) via an aerosol route 30 days after the final boost. Pulmonary bacterial load was assessed using serial dilution of lung homogenates and CFU enumeration on 7H11 agar plates 30 days after challenge. In mice receiving the BCG prime alone, a 0.8 log_10_ CFU reduction in lung bacterial load was observed (Fig. 7), consistent with protective effects of BCG that have been described^40^. Mice primed with BCG and boosted intranasally with co-assembled nanofibers of TB10.4 and Pam2Cys had a significant 1.3 log_10_ CFU reduction in lung bacterial burden compared to naïve mice and an additional 0.5 log_10_ reduction of CFU compared to BCG alone (Fig. 7). Boosting BCG-primed mice with co-assembled nanofibers of TB10.4 and Ag85B did not lead to a discernible decrease in lung bacterial load (Fig. 7) compared to BCG alone. Ag85B is an immunodominant CD4^+^ T helper epitope with established protective efficacy against *Mtb*, which we did not observe here presumably due to the inability of nanofiber adjuvants to activate antigen-presenting cells in the absence of added TLR agonists. Our study compared the efficacy of co-assembled vaccine formulations bearing Pam2Cys or Ag85B in combination with TB10.4. Also, prime-boost with peptide nanofiber vaccines in the absence of BCG failed to confer any protection, suggesting that the cascade of immunological sequela that follow the administration of live-attenuated vaccines is complex and likely activates a broad repertoire of antigen-specific T cells that cannot be replicated using single epitope formulations. Our data strongly indicates that boosting with peptide nanofiber vaccines bearing *Mtb*-specific epitopes and TLR agonists can enhance BCG-primed immunity and reduce lung bacterial load in *Mtb* challenged mice.

## Discussion

Nanomaterials-based vaccine development is gaining increasing momentum due to cross-fertilization of ideas between the fields of nanotechnology, materials science, and immunology. In recent years, joint efforts between bio-engineers and immunologists have led to the development of nanoscale vaccine delivery platforms that have shown considerable promise in small animal models of infectious diseases, cancer, and autoimmunity. Self-assembling peptide nanofibers are one such class of materials that were originally developed for applications in tissue engineering^41,42^, regenerative medicine^43,44^, and drug delivery^45^. However, the last five years have witnessed a growing interest in the application of peptide nanofibers for antigen delivery and immune activation due to their ease of synthesis, self-adjuvanting capacity, and non-inflammatory nature^8,13,15,46^.

In this study, we investigated whether established *Mtb*-specific CD8+ and CD4+ T cell epitopes from immunodominant antigens could be utilized with the peptide nanofiber platform to generate subunit booster vaccines to augment BCG-induced cellular immunity. Multiple studies using peptide libraries encompassing immunogenic *Mtb* proteins (TB10.4, ESAT6, Ag85B) have reported strong protection in preclinical and clinical studies^47–49^. Of the 8 *Mtb* vaccines currently under Phase II/III trials, three are subunit protein vaccines containing one or both of these proteins. TB10.4 is a secretory protein belonging to the early secretory antigenic target gene family known as ESAT6. Studies using human peripheral blood mononuclear cells (PBMCs) from patients with active pulmonary *Mtb*, PPD+ individuals with prior exposure to *Mtb*, or latent *Mtb* infection have identified CD8+ T cell epitopes from TB10.4 that induce strong IFN-γ expression^50^. The low mass secreted ESAT-6 protein is a key molecule recognized by memory effector T cells in a mouse model of long-lived immunity to *Mtb*^51,52^, and Ag85B is the most abundant protein expressed by *Mtb* and has been shown to be efficacious as the primary effector molecule in DNA vaccines, fusion protein vaccines, and liposome-based subunit vaccines^47,53^. We took advantage of the modular nature of self-assembly to co-assemble CD8+ T cell epitopes or CD8+ T cell epitopes in combination with CD4+ T helper epitopes or TLR agonists via simple mixing to generate multivalent formulations that enhance effector and memory recall responses. However our data suggests that co-assembly of antigens does not necessarily lead to synergistic augmentation of the immune response, as evidenced by lower numbers of cytokine-producing CD8+ as well as CD4+ T cells activated by TB10.4 following immunization with co-assembled ESAT6 and TB10.4. While the mechanistic basis for the subdued response to co-assembled antigens is beyond the scope of the current study, a controlled titration of ESAT6 into TB10.4 and Ag85B nanofibers planned for future studies may identify optimal conditions. Further, incorporation of alternative ESAT-6 epitopes with improved immunogenicity could prove valuable. This is important because ESAT6 is a prominent target, which is recognized early during disease in different species infected with *M*. *tuberculosis* or *M*. *bovis*. ESAT6 is also recognized by memory effector T cells in a preclinical model of long-lived immunity to TB and is not present in the vaccine strain *M*. *bovis* BCG.

Natural *Mtb* infection is recognized by the innate immune system through TLR2 and TLR9^54,55^ and vaccine induced activation. Maturation of dendritic cells through TLRs has been shown to contribute significantly to the persistence and magnitude of the immune response^56,57^. Monophosphorylipid A (MPLA, a TLR4 agonist) is currently the only TLR agonist included with licensed vaccines although several innate immune agonists are in various phases of clinical testing specifically in vaccines for *Mtb*^58^. In particular, subunit vaccine design to *Mtb* has involved the use of TLR2 and NOD ligands due to their ability to activate cytotoxic CD8 T cells^59^. TLR2 agonists such as Pam2Cys (a synthetic analog of mycobacterial cell wall component) have been shown to increase antigen uptake, activate dendritic cells and enhance cross-presentation of peptides^60,61^. However, covalent conjugation of Pam2Cys to the self-assembling domain did not diminish the immunostimulatory properties as evidenced by the augmented APC function of DC following exposure to the vaccine construct. In support of optimized T cell activation following boosting, co-assembly of Pam2Cys-KFE8 with TB10.4-KFE8 led to a significant increase in multifunctional CD8+ T cell populations in the lung compared to TB10.4-KFE8 alone, suggesting synergism between nanofiber adjuvants and TLR agonists.

The route of immunization is particularly important when dealing with mucosal pathogens in which immune protection at the site of invasion often correlates with control and clearance or persistence and disease. Mucosally administered vaccines generally induce better mucosal immune responses leading to more efficient protection^62–64^. In the respiratory mucosa, the propagation of T cell-mediated immunity has been shown to be significantly more potent when administered nasally or through aerosol against a host of bacteria including *Mtb* as well as viruses^39,65–67^. Our findings with intranasal administration of peptide nanofiber vaccines also suggests that intranasally delivered vaccines may direct antigen-specific T cell populations to the respiratory mucosa.

The BCG vaccine currently administered to children in over 157 countries prevents several forms of childhood TB, but protection decreases past adolescence proportional to waning of cell-mediated immunity^65^. Booster vaccination strategies based on recombinant adenoviruses^68,69^, cationic liposomes^47^, fusion proteins^70^, as well as synthetic peptides^71^ have been shown to be effective at reinvigorating BCG-primed immunity to *Mtb*. Long-term immunity to many intracellular pathogens is increasingly shown to depend on, or be greatly aided by, development of memory CD8+ T cells. The development of central memory along with effector CD8+ T cells may be useful for assessing the degree to which a vaccine confers protection. Central memory cells defined as CD62L+ CD44 + IL7R^high^KLRG1^low^CD43^low^ were found to be highest in mice that received co-assembled TB10.4-Ag85B nanofibers whereas the percentage of short-lived effector CD8+ T cells (CD62L-CD44 + IL7R^low^KLRG1^high^CD43^high^) and memory precursor effector CD8+ T cells (CD62L-CD44 + IL7R^high^ KLRG1^low^) were comparable between mice receiving TB10.4 nanofibers alone or co-assembled with Ag85B or Pam2Cys (Fig. S3). Further studies are needed to clarify the subsets of lung-resident effector and memory CD8+ T cell phenotypes generated in response to vaccination with peptide nanofiber booster vaccines following a BCG prime. Subunit vaccines are also suitable for immunocompromised individuals such as those with HIV and can prevent further reactivation of those with latent *Mtb* infection. Here we have demonstrated that peptide nanofibers vaccine are promising candidates for boosting BCG-induced cellular immunity and can protect against *Mtb* infection by reducing bacterial load in the lungs of infected mice. Our future studies will seek to evaluate peptide nanofiber vaccines in humanized mouse models of TB^72^ and whole protein antigens conjugated to peptide nanofibers for controlling infection in conjunction with BCG.

## Conclusions

In summary, we have demonstrated that self-assembling peptide nanofibers bearing *Mtb*-specific CD8+ T cell epitopes induce antigen-specific CD8+ T cell responses. Further, multifunctional nanofiber vaccines can be synthesized via simple mixing of CD8+ or CD4+ T cell epitopes or TLR agonists for enhancing the breadth of protection and generating multifunctional T cell populations. In combination with BCG, peptide nanofiber vaccines can reduce bacterial load in the lungs of *Mtb*-infected mice and thus may be an effective strategy for boosting BCG-primed individuals.

## Methods

### Peptide Synthesis

Solid phase synthesis of peptides (Fig. 1) was performed on a CEM Blue (Matthews, NC) microwave synthesizer using standard Fmoc chemistry. All amino acids and resins were purchased from Novabiochem (Billerica, MA). TLR2 agonist (Cys((RS)-2,3-di(palmitoyloxy)-propyl)-OH) was purchased from Bachem (Torrance, CA) and conjugated at the N-terminus of KFE8 to generate TLR2-KFE8 fusion peptide. Peptides were cleaved using cleavage cocktail containing 95% TFA, 2.5% water, and 2.5% triisopropyl silane for 90 min at room temperature and precipitated in ice-cold diethyl ether and purified by reverse phase HPLC using water/acetonitrile gradient on a C18 column. Peptide mass and purity were then assessed by MALDI-TOF mass spectrometry and analytical RP-HPLC. Peptides for immunization were tested for endotoxin using the Lonza LAL QCL-1000™ endotoxin test kit according to the manufacturer’s protocol. None of the peptides tested had detectable levels of endotoxin (<0.1 EU/mL). All peptides were stored at −20 °C until further use.

### Animals and Immunizations

All animal experiments were conducted under approved protocols from the Institution Animal Care and Use Committee at the University of Texas Medical Branch. C57BL6 or BALB/c mice, 6–8 weeks of age were purchased from Jackson labs and housed using conventional methods under appropriate biosafety level containment areas. Mice were housed in microisolator cages with *ad libitum* access to food and water in a standard vivarium for non-infectious studies. Vaccination studies involving use of *M*. *bovis* BCG were performed with animals housed in an animal biosafety level 2 facility, while studies involving *Mtb* were performed within an animal biosafety level 3(ABSL3) facility. Formulations for immunization were prepared fresh as stock solutions (4 mM) in water, incubated overnight at 4 °C, and diluted to working concentration (2 mM) using sterile PBS. The working stocks were incubated at RT for 2 hours prior to inoculation. To prepare co-assembled formulations, the peptides were combined as dry powders, mixed thoroughly, and dissolved in sterile water. The peptides were allowed to incubate overnight at 4 °C and diluted to working concentrations with sterile PBS. Mice were immunized either intranasally or in the left footpad with 25 μL of nanofiber formulations and boosted with 15 μL 30 or 90 days later. 5 days after boost, the draining popliteal and inguinal lymph nodes, or lungs and mediastinal lymph nodes were excised. The lymph nodes were passed through a 70 μM filter and washed twice in sterile cRPMI. Lungs were treated with 1 mg/mL collagenase and 0.5 mg/mL DNase and then homogenized using a gentleMACS dissociator (Miltenyi, USA). The cells were counted and plated onto 96 well plates coated with anti-CD28 at 1 μg/mL in cRPMI (background) or cRPMI containing cognate peptides at 5 μg/mL. Golgiplug™ was added after 1 hour of incubation at 37 °C and the cells were incubated an additional 6 hours to measure cytokine expression. All T-cell stimulation assays were corrected for background cytokine expression by incubation with the irrevelant peptide SIINFEKL or media only. BCG vaccination was performed by subcutaneous inoculation of 10^6^ CFU of *M*. *bovis* BCG (Pasteur strain) in 100 μl of PBS.

### Cell Staining and Flow Cytometry

Cells were stained for extracellular and intracellular markers as described previously^73^ to determine developed of T cell effector, and dendritic cell antigen presenting, function. Briefly, T cell phenotype and function was assessed by washing cells twice in ice cold PBS and stained with a cocktail of anti-CD16/32, anti-CD3 (PerCP-Cy5.5 clone 145-2C11), anti-CD8 (ef450, clone 53-6.7), anti-CD4 (APC-ef780, clone 53-6.7) for 30 minutes at 4 °C. ICS staining was carried out using BD Biosciences fixation and permeabilization kit according BD Biosciences manufacturers recommendations. An ICS staining cocktail of anti-TNF-α (FITC, clone MP6-XT22), anti-IL2 (APC, clone JES6-5H4), anti-IFN-γ (PE-Cy7 clone XMG1.2) was added for 30 min at 4 °C. DC populations were assessed by staining with a cocktail of anti-CD86 (GL1), anti-CD80 (16-10A1), anti-MHC II (M5/114.15.2), and anti-CD11c (N418). Flow cytometric assessment was performed immediately following staining via acquisition on a BD Fortessa LSR-II custom cytometer followed by data analysis using FlowJo software (Ashland, OR).

### Antibody Responses

High-binding ELISA plates (eBioscience) were coated with 20 μg/mL of antigen (KFE8-Ag85B) in PBS overnight at 4 °C and blocked with 200 μL of 1% BSA in PBST (0.5% Tween-20 in PBS) for 1 h. Serum dilutions were applied (1:10^−2^ to 1:10^−9^, 100 μL/well) for 1 h at room temperature followed by addition of peroxidase-conjugated goat anti-mouse IgG (H + L) (Jackson Immuno Research) (1:5000 in 1% BSA-PBST, 100 μL/well). Plates were developed using TMB substrate (100 μL/well, eBioscience), the reaction stopped using 50 μl of 1 M phosphoric acid, and absorbance measured at 450 nm. Absorbance values of PBS (no antigen) coated wells were subtracted to account for background.

### Bioplex Cytokine Assays

To determine if incorporation of TLR agonists into nanofiber formulations augments DC function as an antigen-presenting cell, splenic DCs were isolated and cultured with nanofiber preparations. 100 μL of 5 μg/mL peptide nanofiber solutions of KFE8 and TLR2-KFE8 prepared in sterile PBS were added to 96 well plates and stored at 4 °C overnight. Spleens from 6–8 week old C57/B6 mice were freshly isolated and pushed through a 70 μM filter to create single cell suspensions. RBC Lysis buffer was added and the cells were washed twice in PBS. CD11c+ cells were isolated by positive magnetic selection according to the STEM cell EasySep™ CD11c+ manufacturer’s protocol. Cells were seeded at a density of 500,000 cells/well in cRPMI. Cells were then incubated at 37 °C for 48 hours. The cells were centrifuged and the supernatant was removed for murine 23-factor Bioplex (Biorad USA). Supernatant was diluted 1:2 in PBS and cytokines were measured according to the manufacturers protocol and measured on a Bio-Plex 200 Multiplex System.

### *Mtb* Challenge

Protection from virulent *Mtb* challenge was determined using an aerosol delivery route in order to closely mimic natural infection. An aerosol challenge model was established and tested in both BALB/c and C57BL6 mice (Fig. S4) with assistance from the UTMB Aerobiology Service Center. The Aerobiology Service Center is located within an ABSL3 facility and has the necessary experience and appropriate engineering controls in place for work with risk group 3 (RG3) pathogens such as *Mtb*. *Mtb* (H37Rv strain) was propagated in Middlebrook growth media according to standardized protocols in a Biosafety level 3 laboratory. To prepare inoculum for aerosol delivery, *Mtb* cultures in logarithmic growth phase were diluted to a concentration of 2.92 × 10^5^ CFU/mL in 7H9 media containing x µl/ml anti-foam (Sigma, St. Louis, MO) and placed in the nebulizer. Mice were infected by aerosol inhalation using a whole body exposure box connected to a Biaera Technologies computer-controlled aerosol regulation system housed inside a Class III glove cabinet. The aerosol was generated using a 3-jet Collison nebulizer and aerosol exposures of 10 minutes. The bacterial concentration of the biosampler media within whole body exposure box was used to calculate the presented dose (Dp) of *Mtb* delivered into the lungs of each animal as previously descried^74^. Aerosol delivery of 1 × 10^2^ CFU led to similar pulmonary bacterial loads in non-vaccinated BALB/c and C57BL6 mice 5 wk post-challenge (Fig. S4). Following challenge of vaccinated and control animals; adverse events were monitored daily and changes in body weight determined weekly.

### CFU enumeration

Following aseptic removal at 4 wk post *Mtb* infection lungs were placed into 1 ml of PBS in 15 ml small tissue grinders (Kendall, USA) for tissue homogenization. CFU enumeration was performed using serial dilutions of homogenized organ samples that were prepared in PBS and grown on Middlebrook 7H11 agar plates as described^75^. All studies were performed in a CDC-approved biological safety level-3 (BSL-3) facility.

### Statistical Analysis

All experimental data were plotted using GraphPad Prism software and represented as mean ± SEM, and statistical analysis was performed by ANOVA with multiple comparison of means or Tukey post hoc test. Significance was assigned at p values < 0.05.

## Electronic supplementary material

 Supplementary Information
