# Supplementary material for: Nanoscale Peptide Self-assemblies Boost BCG-primed Cellular Immunity Against Mycobacterium tuberculosis
Source: Sci Rep. 2018 Aug 21;8:12519. doi: 10.1038/s41598-018-31089-y (PMC6104033; doi:10.1038/s41598-018-31089-y)
Supplement: Supplementary file 1 — Supplementary Information [file 41598_2018_31089_MOESM1_ESM.docx]

#### Supplemental Data

***Nanoscale Peptide Self-assemblies Boost BCG-primed Cellular Immunity Against Mycobacterium tuberculosis***

Charles B. Chesson^1^, Matt Huante^2,^ Rebecca J. Nusbaum^3^, Aida G. Walker^2,4^, Tara M Clover^4^, Jagannath Chinnaswamy^5^, Janice J. Endsley^2,6*^ and Jai S. Rudra^4,6*^

^1^Department of Surgical Oncology, Rutgers Cancer Institute of New Jersey, New Brunswick, NJ, 08823, ^2^Department of Microbiology and Immunology, University of Texas Medical Branch, Galveston, TX, 77555, ^3^Department of Pathology, School of Dental Medicine, University of Pennsylvania, Philadelphia, PA, 19104, ^3^, ^4^Department of Pharmacology and Toxicology, ^5^Department of Pathology and Laboratory Medicine, McGovern Medical School, University of Texas Health Science Center at Houston, Houston, TX, 77030, ^6^Sealy Center for Vaccine Development, University of Texas Medical Branch, Galveston, TX 77555


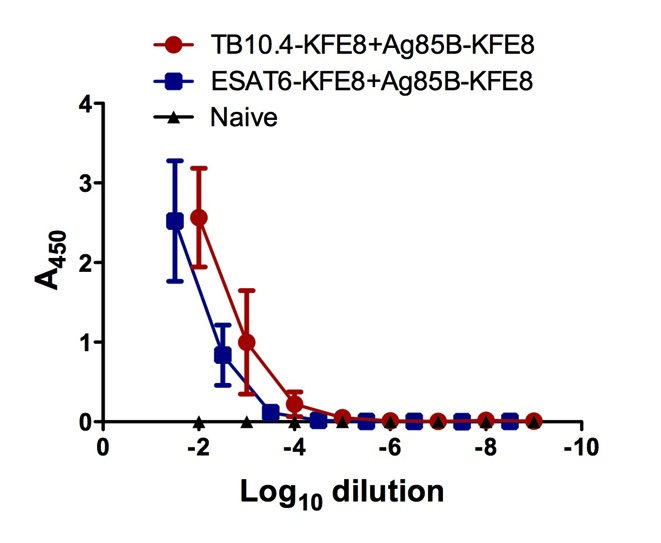


**Figure S1.** Anti-Ag85B antibody production in mice vaccinated with co-assembled nanofiber vaccines of TB10.4-Ag85B or ESAT6-Ag85B. ELISA plates were coated with KF88-Ag85B conjugates and data shows antibody titers (presumably targeted to Ag85B and linker region) over log_10_ serial dilutions of serum starting at 1:100.


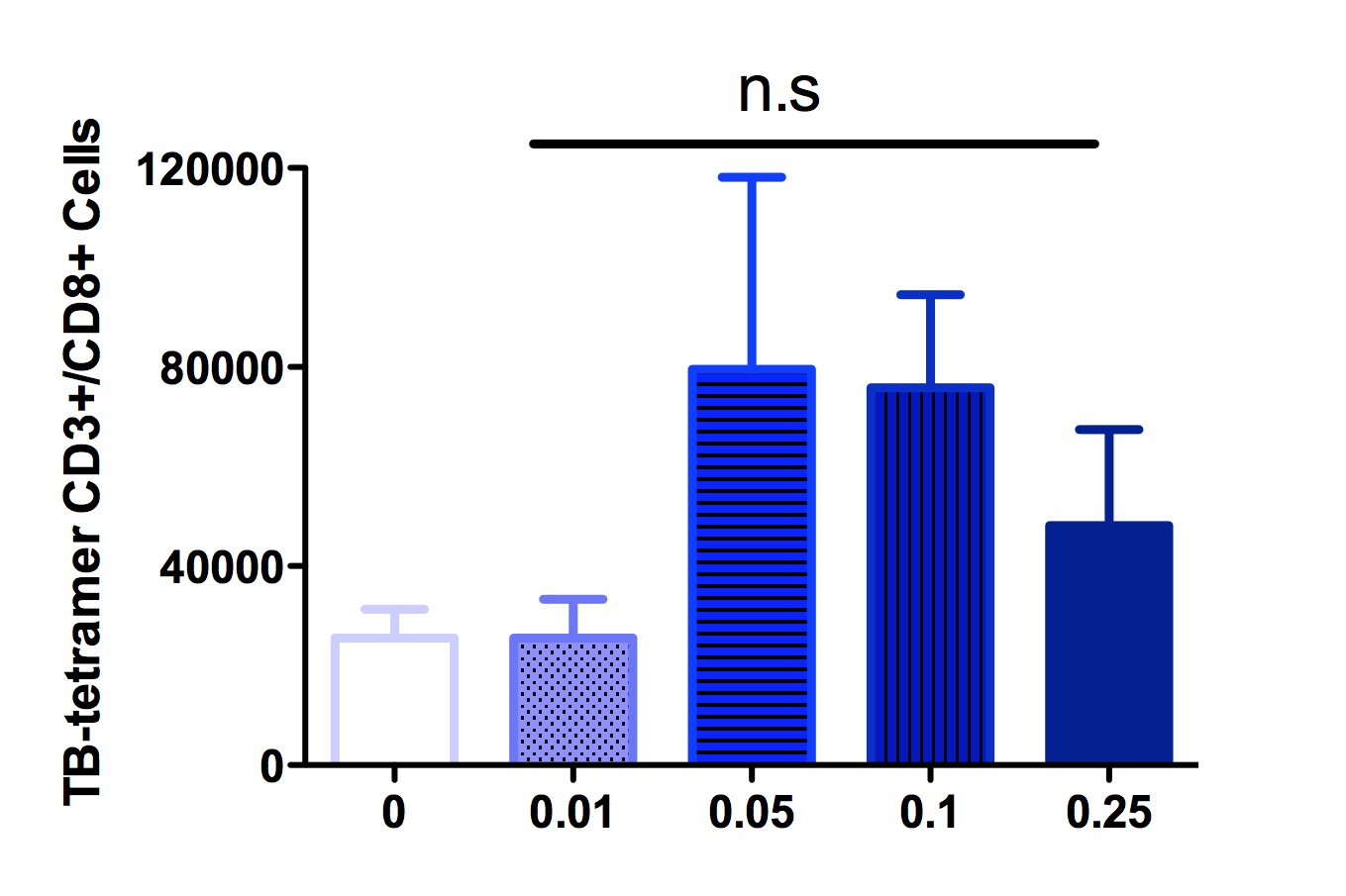


#### Figure S2. Dose response curve showing TB10.4-tetramer positive CD8+T cells in mice vaccinated with co-assembled nanofibers of Pam2Cys-KFE8 and TB10.4-KFE8.

####
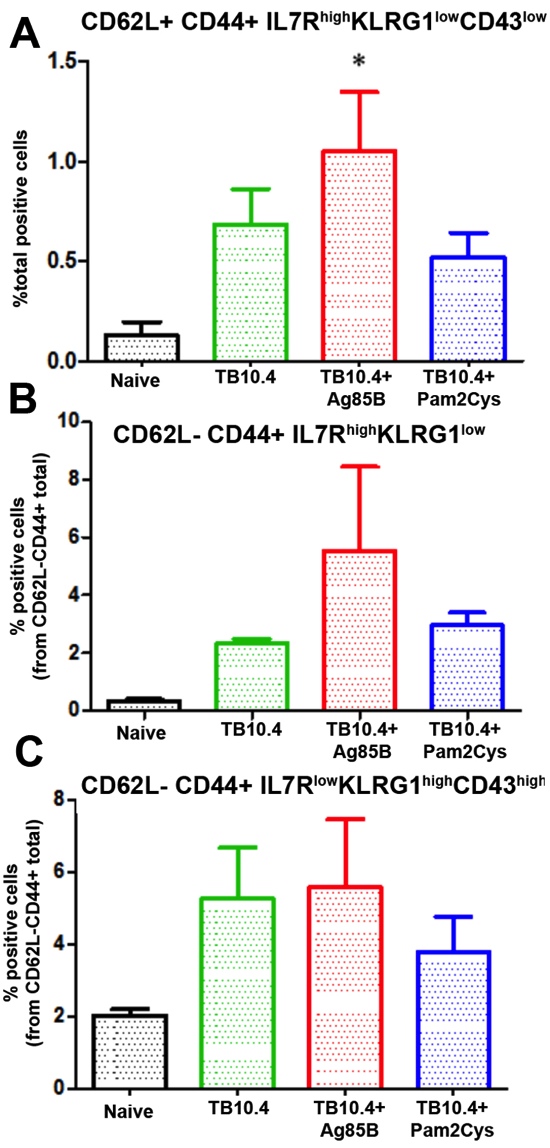


Figure S3. Inclusion of AG85B CD4+T helper cell epitope potentiates development of central memory CD8+T cell populations. C57/B6 mice (N=3-5) were inoculated in the footpad with two doses of nanofiber vaccine separated by 30 days. Total mean percentages of all central memory phenotype CD62L+CD44+IL7R^high^KLRG1^low^CD43^low^ cells from CD3+CD8+CD62L+CD44+ population were found to be highest in mice that received co-assembled TB10.4-Ag85B nanofibers (A). Percentages of memory precursor effector CD8+T cells (CD62L-CD44+IL7R^high^ KLRG1^low^) (B) and short-lived effector CD8+T cells (CD62L-CD44+IL7R^low^KLRG1^high^CD43^high^) (C) were comparable between mice receiving TB10.4 nanofibers alone or co-assembled with Ag85B or Pam2Cys. *p<0.05 by two-way ANOVA with post-hoc analysis for comparison between the means of treatment.


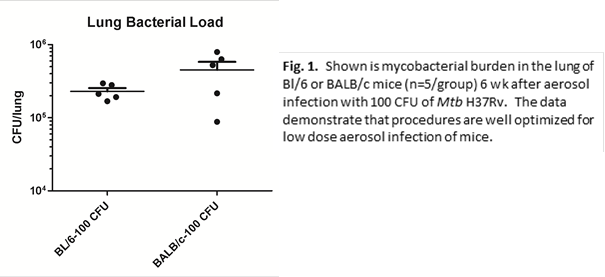


**Figure S4.** Data shows mycobacterial burden in the lungs of C57BL6 or BalbC mice (n=5/group) 6 weeks after aerosol infection with 100 CFU of *Mtb* (H37Rv). The data demonstrate no significant differences in bacterial load between the two different strains and that procedures are well optimized for low dose aerosol infection in mice.


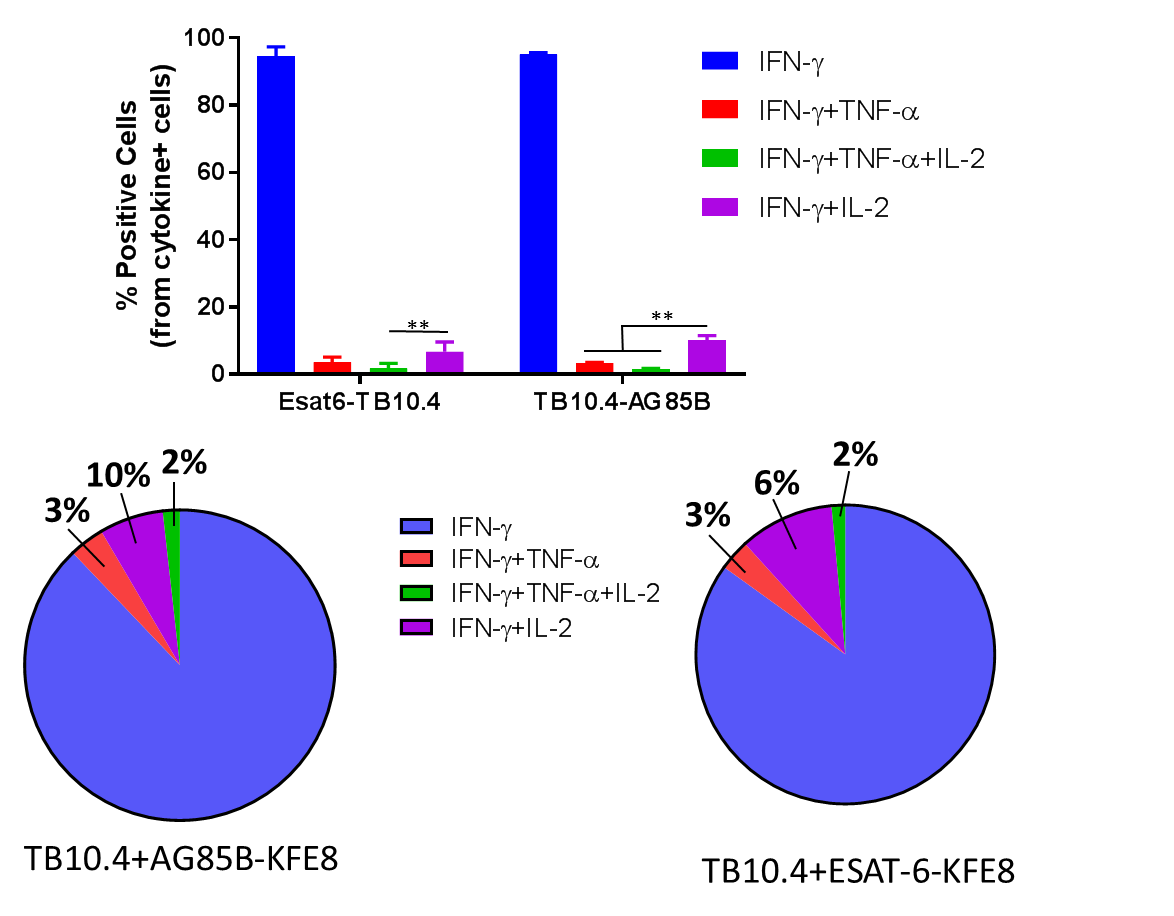


**Figure S5.** Breakdown of the percentage of CD8+CD3+IFN-g+ cells positive for multiple cytokines. These data are an extension of single cytokine positive cells shown in Figure 3 stimulated with the TB10.4 antigen. ESAT6-AG85B co-assembled nanofiber treated samples were not responsive to the TB10.4 antigen (Figure 3).
